# White Spot Syndrome Virus Triggers a Glycolytic Pathway in Shrimp Immune Cells (Hemocytes) to Benefit Its Replication

**DOI:** 10.3389/fimmu.2022.901111

**Published:** 2022-07-04

**Authors:** Yen Siong Ng, Der-Yen Lee, Chun-Hung Liu, Cheng-Yi Tung, Shu-Ting He, Han-Ching Wang

**Affiliations:** ^1^ Department of Biotechnology and Bioindustry Sciences, College of Bioscience and Biotechnology, National Cheng Kung University, Tainan, Taiwan; ^2^ Graduate Institute of Integrated Medicine, China Medical University, Taichung, Taiwan; ^3^ Department of Aquaculture, National Pingtung University of Science and Technology, Pingtung, Taiwan; ^4^ International Center for the Scientific Development of Shrimp Aquaculture, National Cheng Kung University, Tainan, Taiwan

**Keywords:** white spot syndrome virus, white shrimp, glycolysis, stable isotope tracing, hexokinase, phosphofructokinase, lactate dehydrogenase, pyruvate kinase

## Abstract

White spot syndrome virus (WSSV) is the causative agent of a shrimp disease that inflicts in huge economic losses in shrimp-farming industry. WSSV triggers aerobic glycolysis in shrimp immune cells (hemocytes), but how this virus regulates glycolytic enzymes or pathway is yet to be characterized. Therefore, mRNA levels and activity of four important glycolytic enzymes, Hexokinase (HK), Phosphofructokinase (PFK), Pyruvate kinase (PK), and Lactate dehydrogenase (LDH), were measured in WSSV-infected shrimp hemocytes. Gene expression of HK and PFK, but not LDH or PK, was increased at the viral genome replication stage (12 hpi); furthermore, activity of these enzymes, except HK, was concurrently increased. However, there was no increased enzyme activity at the viral late stage (24 hpi). *In vivo* dsRNA silencing and glycolysis disruption by 2-DG further confirmed the role of glycolysis in virus replication. Based on tracing studies using stable isotope labeled glucose, glycolysis was activated at the viral genome replication stage, but not at the viral late stage. This study demonstrated that WSSV enhanced glycolysis by activating glycolytic enzyme at the viral genome replication stage, providing energy and biomolecules for virus replication.

## Introduction

Virus-hijacked metabolism has been investigated for decades to understand how virus alters host metabolic pathways to promote its replication. Metabolic alternation conferred by virus infection commonly resemble metabolic reprogramming in cancer cells (1, 2). By inducing host metabolic pathways, elevated amounts of biomolecules, including nucleotides, amino acids, and lipids, can be subsequently used to produce infectious virion (3). Apart from biomolecule production, virus-induced metabolic reprogramming may also supply ATP in a rapid mode to support energy-intensive processes like viral genome replication and packaging, or NADPH for reductive biosynthesis (lipid synthesis) and maintenance of redox homeostasis (2, 3).

As a carbon source, aerobic glycolysis is generally activated during virus infection to provide ATP, NADPH, and carbon molecules for virus replication. Glycolysis is a compulsory pathway to ensure successful virus replication in viruses that infect vertebrates (4–7). Virus targets rate-limiting glycolytic enzymes, namely Hexokinase (HK), Phosphofructokinase (PFK), and Pyruvate kinase (PK), to control the metabolic rate of glycolysis (1). A viral protein named E4ORF1 from adenovirus induces HK2 and PFKM expression *via* Myc activation to support glycolysis and virus replication (8). Epstein-Barr virus (EBV) oncoprotein LMP1 promotes the transcriptional activity of HK2 *via* c-Myc that upregulates glycolysis (9). Virus not only promotes expression of glycolytic enzymes to increase glycolysis, but can also increase enzyme activity through interactions between viral protein and glycolytic enzyme. For example, by interacting with glycolytic enzyme, hepatitis C virus (HCV) NS5A protein and dengue virus (DENV) NS1 protein boost the activity of HK and GAPDH, respectively (10, 11).

Virus-induced metabolic reprogramming is not confined to cells infected with vertebrate virus, as an invertebrate virus named white spot syndrome virus (WSSV) also reprograms metabolism of its host (shrimp) to facilitate its replication (12, 13). The *in vivo* WSSV replication cycle is ~ 24 h, with viral genome replication stage at 12 hpi, and late stage at 24 hpi (14). At the viral genome replication stage (12 hpi), WSSV triggers several metabolic pathways, e.g., aerobic glycolysis, pentose phosphate pathway, nucleotide biosynthesis, glutaminolysis, lipolysis, and amino acid biosynthesis, in shrimp immune cells (hemocytes) and other target tissues (12–17).

To complete virus replication, WSSV induces aerobic glycolysis in shrimp hemocytes at the viral genome replication stage (12 hpi) (14). The glycolytic shift was accommodated by regulation of glycolytic enzymes (13, 18–20). Godoy-Lugo et al. (2019) reported that transcriptional factor HIF-1 regulates HK, PFK, and PK in a tissue-specific manner in WSSV-infected white shrimp (19). In ridgetail white prawn (*Exopalaemon carinicauda*), WSSV increased expression of HK and PFK (20). In WSSV infection, low activity of pyruvate dehydrogenase (PDH) diverts glucose into lactate production instead of entering the TCA cycle (13, 18). Lactate dehydrogenase, which catalyzes the conversion of pyruvate to lactate, is activated during WSSV infection (21). As most pyruvate is shunted into lactate, glutamate anaplerosis sustains the TCA cycle, facilitating ongoing production of energy and biomolecules (17). Additionally, Glucose-6-phosphate dehydrogenase (G6PDH) is activated during WSSV infection, redirecting glucose-6-phosphate, a glycolytic intermediate, into the pentose phosphate pathway (14). This not only promotes nucleotide biosynthesis for virus replication, but also produces enough NADPH, a reducing agent, to neutralize ROS produced during virus infection (13, 18).

As an important pathway for virus replication, glycolysis has been investigated in WSSV-infected shrimp; however, most studies have only characterized an individual glycolytic enzyme. An isotope-labelling tracing of glucose in WSSV-infected shrimp could provide a comprehensive understanding of glycolysis during WSSV infection. In this study, stable isotope-labeled glucose ([U-^13^C] Glucose) was used as a tracer to track various glycolytic metabolites during WSSV infection. Four important glycolytic enzymes (HK, PFK, PK and LDH), were investigated to assess their roles in WSSV infection.

## Materials and Methods

### Experimental Animals and WSSV Inoculum

Juvenile white shrimp (*Litopenaeus vannamei*, 2~3 g) used in this study were obtained from the International Center for the Scientific Development of Shrimp Aquaculture, National Cheng Kung University (NCKU) and the Department of Aquaculture, National Pingtung University of Science and Technology (NPUST). Shrimp were kept in 30 ppt sterilized seawater at 27°C for 1 d before virus infection. The WSSV stock (Taiwan isolate, GenBank accession no. AF440570) was prepared by collecting hemolymph of WSSV-infected moribund SPF shrimp, as described (13). The WSSV stock was diluted 10^-4^ with 1x PBS (137 mM NaCl, 2.7 mM KCl, 10 mM Na_2_HPO_4_, and 2 mM KH_2_PO_4_) and used for intramuscular injection into shrimp. The WSSV challenge dosage (100 μl/3 g shrimp) induced ~50% mortality in 3 d and 100% in 5 d, whereas PBS-treated shrimp (100 μl/3 g shrimp) served as a control. At 12 and 24 h after WSSV challenge, hemocytes were collected to assess enzyme activity and quantify expression of glycolytic and WSSV genes. Pleopods were collected to measure WSSV genome copy numbers.

### Quantification of Glycolytic Genes and WSSV Structural Gene VP28 by Real-Time PCR

Extraction of RNA from hemocytes collected 12 and 24 hpi was done with REzol (Protech Enterprise) and cDNA synthesized with SuperScript™ II Reverse Transcriptase (Invitrogen) and Anchor-dTv primer (**Table 1**). The resulting cDNA was used to measure expression of target genes, using the Bio-Rad detection system and KAPA SYBR^®^ FAST qPCR Master Mix (KAPA). Primer sets of each target gene are listed (**Table 1**). Data were normalized with the values of EF-1α (internal control) and calculated by the 2^-ΔCT^ method. The empirical rule was performed on all data for detection and exclusion of statistical outliers. Differences between groups were determined with a Student’s t-test, as described (22).

**Table 1:** Primer sets used in the present study.

Gene^a^	Primer	Primer sequence (5’-3’)^b^	Function
HK (PVHP142913.2)			
	HK-ds-F1	5’-GAAATCTGCCAGGAGCTG-3’	Cloning
	HK-ds-R1	5’-GTGCCTGTAGTGTCATTCAG-3’	Cloning
	T7-HK-ds-F1	5’-TAATACGACTCACTATAGGGAGAGAAATCTGCCAGGAGCTG-3’	dsRNA synthesis
	T7-HK-ds-R1	5’-TAATACGACTCACTATAGGGAGAGTGCCTGTAGTGTCATTCAG-3’	dsRNA synthesis
	HK-qF	5’-GACCTGGTGATGGGCTGTTT-3’	Real-time PCR
	HK-qR	5’-GTTCCGTTCCTTGATGAAGCTT-3’	Real-time PCR
PFK (PVHP206412.2)			
	PFK-ds-F1	5’-CTGCAGTTCGTGCTGTAGT-3’	Cloning
	PFK-ds-R1	5’-CGTAATCAGCCTCAGAGGTC-3’	Cloning
	T7-PFK-ds-F1	5’-TAATACGACTCACTATAGGGAGACTGCAGTTCGTGCTGTAGT-3’	dsRNA synthesis
	T7-PFK-ds-R1	5’-TAATACGACTCACTATAGGGAGACGTAATCAGCCTCAGAGGTC-3’	dsRNA synthesis
	PVHP206412.2-qF	5’-AAGGGTGGCACAGTCATTGG-3’	Real-time PCR
	PVHP206412.2-qR	5’-TCACGGTCGCGGAAATCT-3’	Real-time PCR
LDH (PVHP240876.1)			
	LDH-ds-F1	5’-GGTTGATGTTGCTGCTGAC-3’	Cloning
	LDH-ds-R1	5’-TCAGGATCTTCTGGAGTTCC-3’	Cloning
	T7-LDH-ds-F1	5’-TAATACGACTCACTATAGGGAGAGGTTGATGTTGCTGCTGAC-3’	dsRNA synthesis
	T7-LDH-ds-R1	5’-TAATACGACTCACTATAGGGAGATCAGGATCTTCTGGAGTTCC-3’	dsRNA synthesis
	PVHP240876.1-qF	5’-CCCCAAGCACCATGTGATC-3’	Real-time PCR
	PVHP240876.1-qR	5’-GAAGCGGAATCTGGCAGAGT-3’	Real-time PCR
PK (PVHP133145.4)			
	PK-ds-F1	5’-CAAGTTGACCACAGATGCC-3’	Cloning
	PK-ds-R1	5’-TTCCAACATCTGGGTAGCAC-3’	Cloning
	T7-PK-ds-F1	5’-TAATACGACTCACTATAGGGAGACAAGTTGACCACAGATGCC-3’	dsRNA synthesis
	T7-PK-ds-R1	5’-TAATACGACTCACTATAGGGAGATTCCAACATCTGGGTAGCAC-3’	dsRNA synthesis
	PVHP133142.1-qF	5’-GGACCTGTCTCTCGGTCTGTAGA-3’	Real-time PCR
	PVHP133142.1-qR	5’-TGTTCATGCCAGCCTCCAT-3’	Real-time PCR
EF-1α			
	EF1α-F	5’-ATGGTTGTCAACTTTGCCC-3’	Cloning
	EF1α-R	5’-TTGACCTCCTTGATCACACC-3’	Cloning
	EF1α-qF	5’-ACGTGTCCGTGAAGGATCTGAA-3’	Real-time PCR
	EF1α-qR	5’-TCCTTGGCAGGGTCGTTCTT-3’	Real-time PCR
Luciferase			
	Luc-F	5’-CTGAATACAAATCACAGAATC-3’	Cloning
	Luc-R	5’-GCGAGAATCTGACGCAGGCAGT-3’	Cloning
	T7-Luc-F	5’-TAATACGACTCACTATAGGGAGACTGAATACAAATCACAGAATC-3’	Real-time PCR
	T7-Luc-R	5’-TAATACGACTCACTATAGGGAGAGCGAGAATCTGACGCAGGCAGT-3’	Real-time PCR
VP28			
	VP28-real-F	5’-AGTTGGCACCTTTGTGTGTGGTA-3’	Real-time PCR
	VP28-real-R	5’-TTTCCACCGGCGGTAGCT-3’	Real-time PCR
Others			
	Anchor-dTV	5’-GACCACGCGTATCGATGTCGACTTTTTTTTTTTTTTTTV-3’	cDNA synthesis

^a^Each primer set for glycolytic gene was designed using an in-house transcriptomic database, with the accession number written beside its corresponding gene.

^b^The added T7 promoter sequence is underlined.

### WSSV Genome Copy Numbers

Extraction of genomic DNA from pleopods collected 24 hpi was done with a DTAB/CTAB DNA extraction kit (GeneReach Biotechnology Corp.) and WSSV genome copy numbers were determined with an IQ Real™ WSSV quantitative system (GeneReach Biotechnology Corp.). Differences between groups were detected with a Student’s t-test, as described above.

### Hexokinase (HK) Activity in WSSV-Infected Shrimp Hemocytes

Hemocytes collected at 12 and 24 hpi (6 shrimp/pool and 4 pools/group) were used to assess hexokinase activity, with a hexokinase colorimetric assay kit (Biovision). Hemocytes were homogenized with 100 µl HK assay buffer. The homogenate was incubated on ice for 10 min and centrifugated at ~13,000 x g for 5 min. Protein concentration in the supernatant was determined by a Bradford assay (Bio-Rad Protein Assay Dye Reagent Concentrate). 5 μg of hemocyte protein was added to a 96 well plate, with addition of 50 μl HK assay buffer. The NADH standard curve was prepared along with the sample group. The reaction mixture was later added to start the reaction (34 µl HK assay buffer, 2 µl HK enzyme mix, 2 µl HK developer, 2 µl HK coenzyme, and 10 µl HK substrate). Background controls were prepared as per the samples except that HK substrate was not added. Absorbance was determined at 450 nm and room temperature every 2 min for 50 min, with NADH production calculated using the standard curve. Sample HK activity was calculated by the following equation: (B2-B1)/(ΔT x P), where B2 is the NADH produced from the sample (nmol) at the time of second reading; B1 is the NADH amount produced from the sample (nmol) at the time of first reading; ΔT is the reaction time between first and second reading (min); and P is the added protein amount. Differences between groups were detected by Student’s t-test.

### Phosphofructokinase (PFK) Activity in WSSV-Infected Shrimp Hemocytes

A phosphofructokinase activity colorimetric assay kit (Biovision) was used to measure PFK activity. Hemocytes collected at 12 and 24 hpi (6 shrimp/pool and 4 pools/group) were homogenized with 100 µl PFK assay buffer. The homogenate was centrifuged at ~13,000 x g for 5 min and a Bradford assay done to quantify protein concentration. Then, 1.25 µg hemocyte protein was added to a 96 well plate, and 50 µl PFK assay buffer added. Reaction was initiated at 37°C by adding reaction mixture containing 42 µl PFK assay buffer, 2 µl PFK enzyme mix, 2 µl PFK developer, 2 µl ATP and 2 µl PFK substrate. Background controls were prepared as per the samples except that PFK substrate was not added. The NADH standard curve was prepared along with the sample group. The total mixture was read at 450 nm each minute for 40 min. The sample PFK activity was calculated as follows: (B2-B1)/(ΔT x P), with a Student’s t-test used to detect differences.

### Pyruvate Kinase (PK) Activity in WSSV-Infected Shrimp Hemocytes

In hemocytes collected at 12 and 24 hpi (6 shrimp/pool and 4 pools/group), PK activity was determined with a pyruvate kinase activity colorimetric/fluorometric assay kit (Biovision). Hemocytes were homogenized with 100 µl PK assay buffer, and cell debris removed by centrifuging the homogenate (10,000 x g for 1 min). After the protein concentration was determined, 2.5 μg of hemocyte protein was added to a 96 well plate, followed by 50 μl of PK assay buffer. A pyruvate standard curve was also prepared. Reaction was initiated at room temperature by adding 44 μl PK assay buffer, 2 μl Substrate mix, 2 μl Enzyme mix, and 2 μl OxiRed™ Probe Background controls were prepared as per the samples except that the substrate mix was not added. Activity was measured at 570 nm every minute for 20 min and activity was calculated as follows: (B2-B1)/(ΔT x P). Student’s t-test was used to detect differences.

### Lactate Dehydrogenase (LDH) Activity in WSSV-Infected Shrimp Hemocytes

The LDH activity in hemocytes collected at 12 and 24 hpi (6 shrimp/pool and 4 pools/group) was assessed with a lactate dehydrogenase activity colorimetric assay kit (Biovision). After hemocytes were homogenized in 150 μl LDH assay buffer, the homogenate was centrifuged at 10 000 x g for 15 min at 4°C and protein concentration in the supernatant quantified by Bradford assay. Then, 10 μg of hemocyte protein was brought to a final volume of 50 μl with LDH assay buffer. Reaction was initiated at 37°C by adding 48 μl LDH assay buffer and 2 μl substrate mix solution and activity determined at 450 nm every 2 min for 30 min. The activity was calculated with the following calculation: (B2-B1)/(ΔT x P). Student’s t-test was used to detect differences.

### *In Vivo* Gene Silencing of Glycolytic Enzyme by dsRNA Interference

An in-house *L. vannamei* stomach transcriptomic database established with next generation sequencing (data not shown) was used to design primer sets for each glycolytic gene. Sequences of the primer sets are listed in **Table 1**. Partial sequences of HK, PFK, PK, LDH, and luciferase control were amplified using PCR and corresponding primer sets: HK-ds-F1/HK-ds-R1, PFK-ds-F1/PFK-ds-R1, LDH-ds-F1/LDH-ds-R1, PK-ds-F1/PK-ds-R1, and Luc-F/Luc-R. The T7 promoter sequence was then incorporated into the amplicon by PCR, using the following primer sets: HK: T7-HK-ds-F1/HK-ds-R1 and HK-ds-F1/T7-HK-ds-R1; PFK: T7-PFK-ds-F1/PFK-ds-R1 and PFK-ds-F1/T7-PFK-ds-R1; LDH: T7-LDH-ds-F1/LDH-ds-R1 and LDH-ds-F1/T7-LDH-ds-R1; PK: T7-PK-ds-F1/PK-ds-R1 and PK-ds-F1/T7-PK-ds-R1; Luc: T7-Luc-F/Luc-R and Luc-F/T7-Luc-R. A T7-anchored amplicon was used to generate ssRNA by using T7 RiboMax™ express large scale RNA production system kit (Promega). Two complementary ssRNA were incubated together to synthesize dsRNA, which was purified by phenol/chloroform extraction. The dsRNA products were quantified by UV spectrophotometer, verified with agarose gel electrophoresis and stored at -80°C.

Shrimp (~3 g body weight) were injected with the synthesized dsRNA (diluted with 0.22 µm-filtered PBS, 1 μg/g shrimp) 3 d before virus injection. Luciferase dsRNA or PBS served as controls. At 72 h post dsRNA injection, some hemocytes were collected (3 shrimp/pool, 4 pools/group) to confirming the efficiency of gene silencing, whereas the remaining shrimp were injected with virus inoculum or used as a control.

### Stable Isotope-Labeled Glucose Tracer and Liquid Chromatography Electrospray Ionization Mass Spectrometry (LC-ESI-MS) To Monitor Metabolites

**Figure 1:**
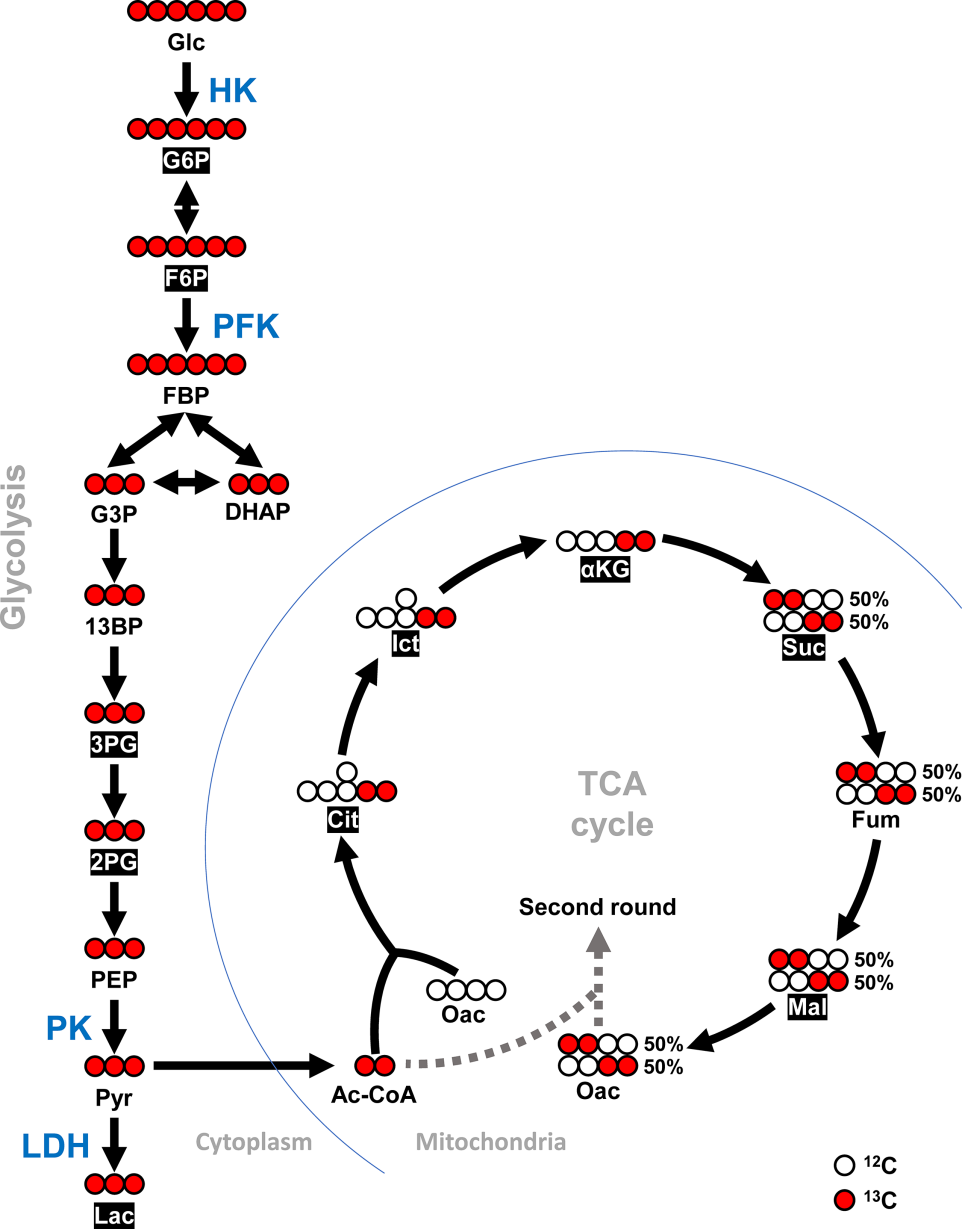
Schematic diagram of [U-^13^C] glucose entering TCA cycle *via* glycolysis. Detected ^13^C metabolites in this study are shown in white font within black boxes. Red circles represent carbon-13 (^13^C) and white circles represent carbon-12 (^12^C). This diagram was adapted and modified from McDonald et al. (23) and Courtney et al. (24). For metabolites, Glc, Glucose; G6P, Glucose-6-phosphate; F6P, Fructose-6-phosphate; FBP, Fructose 1,6-biphosphate; G3P, Glyceraldehyde-3-phosphate; DHAP, Dihydroxyacetone phosphate; 13BP, 1,3-Bisphosphoglycerate; 3PG, 3-Phosphoglycerate; 2PG, 2-Phosphoglycerate; PEP, Phosphoenolpyruvate; Pyr, Pyruvate; Lac, Lactate; Ac-CoA, Acetyl-CoA; Cit, Citrate; Ict, Isocitrate; αKG, α-Ketoglutarate; Suc, Succinate; Fum, Fumarate; Mal, Malate; and Oac, Oxaloacetate. For enzymes, HK, Hexokinase; PFK, Phosphofructokinase; PK, Pyruvate kinase; and LDH, Lactate dehydrogenase.

The WSSV-infected shrimp were injected with [U-^13^C] glucose to facilitate tracing the stable carbon isotope through glycolysis (**Figure 1**). The procedure was done as described (15). Briefly, the stable isotope-labeled [U-^13^C] glucose (Cambridge Isotope Laboratories Inc., USA) was injected into the abdominal hemal sinus (450 μg/g shrimp) at 12 or 24 hpi. At 10 or 30 min after tracer injection, hemocyte samples (3 shrimp/pool, 4 pools/group) were collected and MeOH used to extract metabolites, which were lyophilized and subjected to LC-ESI-MS analyses.

Ultra-performance liquid chromatography (UPLC) system (Ultimate 3000 RSLC, Dionex) and a quadrupole time-of flight (QTOF) mass spectrometer with an electrospray ionization (ESI) source (maXis HURQToF system, Bruker Daltonics) were used for LC-ESI-MS analyses. The metabolites sample was dissolved in ddH_2_O, reaction buffer (0.3 M aniline [Sigma-Aldrich, USA] in 60 mM HCl), and N-(3-dimenthylaminopropyl)-N’-ethylcarbodiimide hydrochloride (EDC; Sigma-Aldrich, USA) and incubated for 2 h at 25°C, with 10% ammonium hydroxide added to stop the reaction. The derivatives were subjected to reversed-phase liquid chromatography (RPLC) with a BEH C18 column (2.1 x 100 mm, Waters). The elution initiated from 99% mobile phase A (0.1% formic acid in ddH_2_O) and 1% mobile phase B (0.1% formic acid in ACN), held at 1% B for 0.5 min, raised to 60% B in 6 min, further raised to 90% B in 0.5 min, held at 90% B for 1.5 min, and then lowered to 1% B in 0.5 min; then, 1% B was used to equilibrate the column for 4 min. The injection volume was 10 µl and flow rate was 0.3 ml/min. The LC-ESI-MS chromatograms were obtained under a capillary voltage of either 4,500 or 3,500 V in negative ion mode, a dry temperature of 190°C, a dry gas flow maintained at 8 l/min, nebulizer gas at 1.4 bar, and an acquisition range of 100-1,000 m/z.

HyStar and micrOTOF control software (Bruker Daltonics) were used to obtain the data, which were assessed with DataAnalysis and TargetAnalysis software (Bruker Daltonics). To monitor changes in quantities of ^13^C labeled metabolites, fold changes in the WSSV group were calculated relative to the corresponding PBS group (WSSV/PBS group). All signal counts were normalized by the sample weight and differences between groups were analyzed by Student’s t-test.

### Effects of 2-Deoxy-D-Glucose (2-DG) on WSSV Replication

To investigate involvement of glycolysis in WSSV replication, 2-DG (Sigma), a structural analogue of glucose, was used to disrupt glycolysis in WSSV-infected shrimp. Shrimp were injected with 100 μl 2-DG solution (dissolved in 0.22 µm-filtered PBS, 0.5 mg/g shrimp) twice before the WSSV infection (at 1 d and at 2 h before virus infection), with PBS-injected shrimp as a control. Sample collection was done 24 hpi and subjected to WSSV structural gene (VP28) expression and WSSV genome copy numbers quantification.

## Results

### Hexokinase (HK) Was Required for WSSV Replication

**Figure 2:**
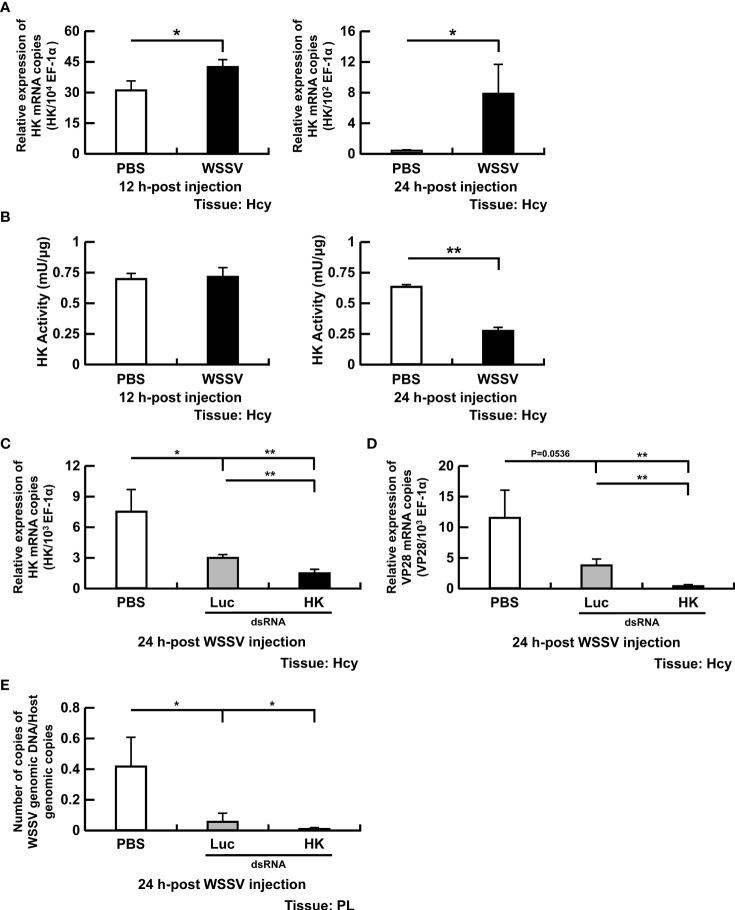
Participation of HK in WSSV replication. **(A, B)** The mRNA levels and enzyme activity of HK in shrimp hemocytes during WSSV infection. **(C)** For HK dsRNA silencing, gene expression of HK in shrimp hemocytes was analyzed by real-time PCR at 24 h post WSSV injection. **(D, E)** Effects of gene silencing of HK on expression of the WSSV structural gene VP28 and WSSV genome copy numbers at 24 h post WSSV injection. Groups treated with PBS only or with non-specific luciferase (Luc) dsRNA were used as control groups. WSSV genome copy numbers was 484-fold decreased in HK dsRNA group in relative to Luc dsRNA group. Each bar represents the mean ± SD. Asterisks indicate differences between indicated groups (*p < 0.05; **p < 0.01). Hcy: Hemocytes; and PL, Pleopods.

In this study, four important glycolytic enzymes, HK, PFK, PK and LDH, were analyzed (**Figure 1**). Hexokinase catalyzes the first step of glycolysis, phosphorylating glucose to glucose-6-phosphate. In WSSV-infected shrimp, HK gene expression was increased at 12 and 24 hpi (**Figure 2A**). Regarding HK activity, there was no change in hemocytes of WSSV-infected versus PBS-treated shrimp at 12 hpi; however, at 24 hpi, activity was decreased in WSSV-infected shrimp (**Figure 2B**).

To further investigate the importance of HK in WSSV replication, dsRNA-mediated *in vivo* silencing of HK was done. At 72h-post HK dsRNA treatment, a large number of shrimp deaths were observed. Surviving shrimp were injected with WSSV and hemocytes were collected 24 h later. The mRNA expression of HK was significantly suppressed by HK dsRNA at 24 hpi compared to PBS or luciferase control (**Figure 2C**). Furthermore, WSSV VP28 mRNA expression (**Figure 2D**) and WSSV genome copy numbers (**Figure 2E**) were decreased in WSSV-injected shrimp pretreated with HK dsRNA.

### Phosphofructokinase (PFK) and Lactate Dehydrogenase (LDH) Were Required for WSSV Replication

**Figure 3:**
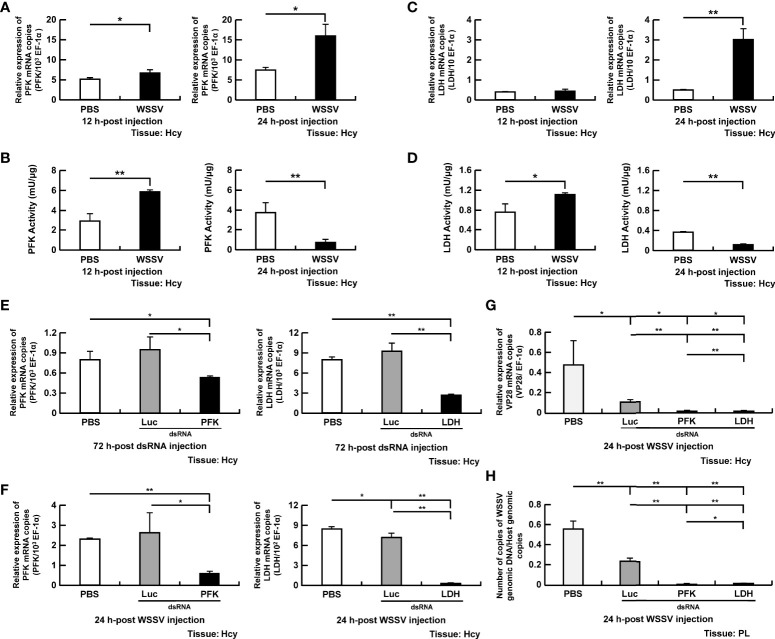
Participation of PFK and LDH in WSSV replication **(A, B)** The mRNA levels and enzyme activity of PFK in shrimp hemocytes during WSSV infection. **(C, D)** The mRNA levels and enzyme activity of LDH in shrimp hemocytes during WSSV infection. **(E)** For PFK and LDH dsRNA silencing, gene expression of PFK and LDH in shrimp hemocytes was analyzed by real-time PCR at 72 h post injection of the corresponding dsRNA and before WSSV challenge. **(F)** Gene expression of the above genes was measured again in dsRNA-treated shrimp at 24 h post WSSV infection. **(G H)** The effect of gene silencing of PFK and LDH on the expression of the WSSV structural gene VP28 and WSSV genome copy numbers at 24 h post WSSV injection. Groups treated with PBS only or with non-specific luciferase (Luc) dsRNA were used as control groups. WSSV genome copy numbers were 79-fold and 18-fold decreased in PFK and LDH dsRNA group respectively, compared to Luc dsRNA group. Each bar represents the mean ± SD. Asterisks indicate differences between the indicated groups (*p < 0.05; **p < 0.01). Hcy, Hemocytes, PL, Pleopods.

Phosphofructokinase, a rate-limiting enzyme in glycolysis, promotes phosphorylation of fructose-6-phosphate to fructose 1,6-bisphosphate. At 12 and 24 hpi, PFK gene expression was increased in WSSV-infected shrimp compared to the PBS controls (**Figure 3A**). The PFK activity was increased in WSSV-infected hemocytes compared to PBS-treated hemocytes at 12 hpi, whereas at 24 hpi, PFK activity was significantly reduced (**Figure 3B**).

Lactate dehydrogenase, which converts the pyruvate to lactate, was also selected for the study. Although LDH gene expression was unchanged in WSSV-infected shrimp at 12 hpi (**Figure 3C**), there was increased LDH activity in hemocytes (**Figure 3D**). At 24 hpi, gene expression of LDH was significantly increased in the WSSV infection group (**Figure 3C**), but activity was decreased in hemocytes (**Figure 3D**).

To further explore the role of PFK and LDH in WSSV replication, shrimp were treated with the corresponding dsRNA to suppress gene expression. Gene expression of PFK and LDH were suppressed in the corresponding dsRNA-treated shrimp before virus infection and at 24 hpi (**Figures 3E, F**). Both PFK and LDH silencing significantly decreased WSSV VP28 mRNA expression (**Figure 3G**) and WSSV genome copy numbers (**Figure 3H**).

### Pyruvate Kinase (PK) Was Required for WSSV Replication

**Figure 4:**
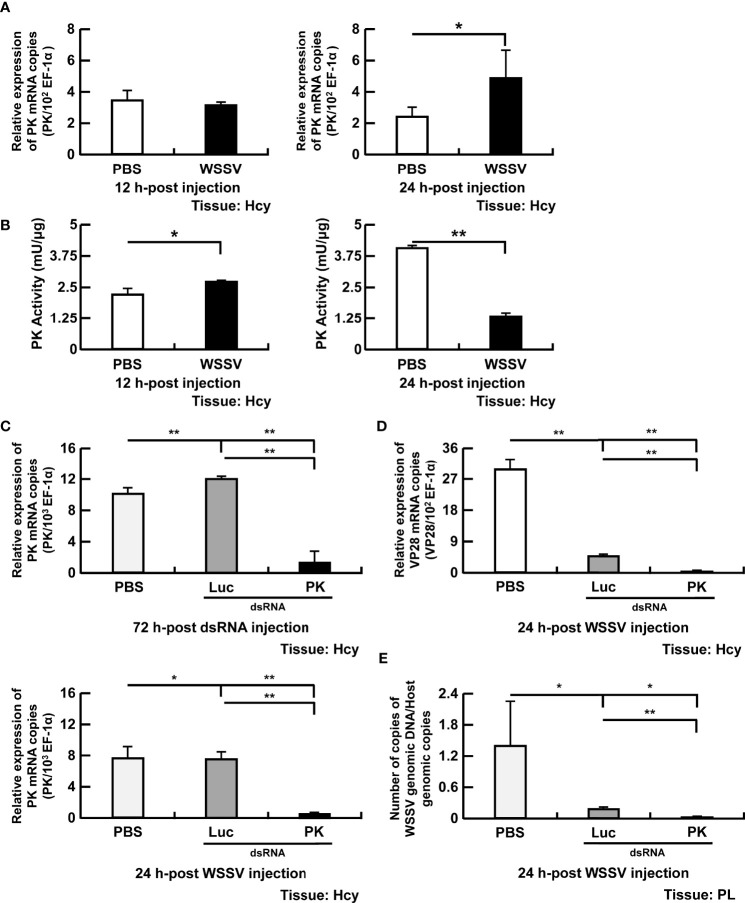
Participation of PK in WSSV replication. **(A, B)** The mRNA levels and enzyme activity of PK in shrimp hemocytes during WSSV infection. **(C)** For PK dsRNA silencing, gene expression of PK in shrimp hemocytes was analyzed by real-time PCR at 72 h post injection of the PK dsRNA and before WSSV challenge. PK gene expression was measured again in dsRNA-treated shrimp at 24 h post WSSV injection. **(D, E)** Effects of gene silencing of PK on expression of the WSSV structural gene VP28 and WSSV genome copy numbers at 24 h post WSSV injection. Groups treated with PBS only or with non-specific luciferase (Luc) dsRNA were used as control groups. WSSV genome copy numbers was 225-fold decreased in PK dsRNA group in relative to Luc dsRNA group. Each bar represents the mean ± SD. Asterisks indicate differences between the indicated groups (*p < 0.05; **p < 0.01). Hcy, Hemocytes; and PL, Pleopods.

Pyruvate kinase is a rate-limiting enzyme that catalyzes the final step of glycolysis by converting phosphoenolpyruvate to pyruvate. Expression of PK was unchanged in WSSV-infected shrimp at 12 hpi, whereas a significant increase of expression occurred at 24 hpi (**Figure 4A**). In contrast with gene expression, PK activity was elevated in WSSV-infected hemocytes at 12 hpi, but reduced at 24 hpi (**Figure 4B**). After PK dsRNA treatment, low expression of PK in the silencing group indicated that PK dsRNA-mediated gene silencing was successful (**Figure 4C**). Silencing PK significantly reduced both WSSV VP28 mRNA expression (**Figure 4D**) and WSSV genome replication (**Figure 4E**).

### *In Vivo* Tracking of [U-^13^C] Glucose-Derived Metabolites Revealed Glycolysis Was Activated at the Viral Genome Replication Stage (12 hpi)

**Figure 5:**
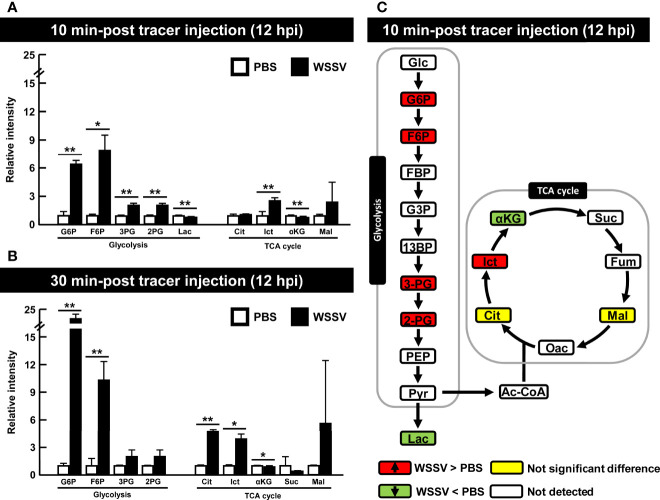
WSSV activated glycolysis at the viral genome replication stage (12 hpi). At 12h after challenge with WSSV or PBS, shrimp were injected with [U-^13^C] glucose and hemocytes were collected after **(A)** 10 min or **(B)** 30 min of tracer injection. Metabolomic data were generated with LC-ESI-Q-TOF-MS. Fold change of each ^13^C metabolites in WSSV group compared to the corresponding ^13^C metabolites in PBS group was calculated. Each bar represents the mean ± SD. Asterisks indicate differences between WSSV and PBS groups (*p < 0.05; **p < 0.01). **(C)** Overview of changes of ^13^C metabolites in WSSV-infected shrimp (12 hpi) at 10 min post [U-^13^C] glucose injection. Changes in the WSSV group relative to the corresponding PBS control were rated as a significant increase (Red), no significant difference (Yellow), a significant decrease (Green), or not detected (White). Abbreviations are the same as those used in **Figure 1**.

Shrimp infected with WSSV were injected with [U-^13^C] glucose at 12 hpi to investigate glycolytic metabolite during WSSV replication. Specifically, the carbon-13 (^13^C) from the labeled [U-^13^C] glucose was tracked through the glycolysis pathway and the TCA cycle as it was transferred to downstream metabolites during enzymatic activity (**Figure 1**). Although the level of labeled carbon in several metabolites was undetectable, there was a notable increase in the glycolytic metabolites glucose-6-phosphate (G6P), fructose-6-phosphate (F6P), 3-phosphoglycerate (3-PG), and 2-phosphoglycerate (2-PG) in the WSSV group at 10 min after tracer injection, while in the TCA cycle, isocitrate (Ict) was elevated and α-ketoglutarate (α-KG) was reduced (**Figure 5A**). At 30 min after tracer injection, both citrate (Cit) and isocitrate (Ict) were elevated (**Figure 5B**). Metabolite changes 10 min after tracer injection (at 12 hpi) are summarized in **Figure 5C**, and raw metabolomic data are provided in **Table S1**.

### *In Vivo* Tracking of [U-^13^C] Glucose-Derived Metabolites Suggested Glycolysis Was Not Activated at Viral Late Stage (24 hpi)

**Figure 6:**
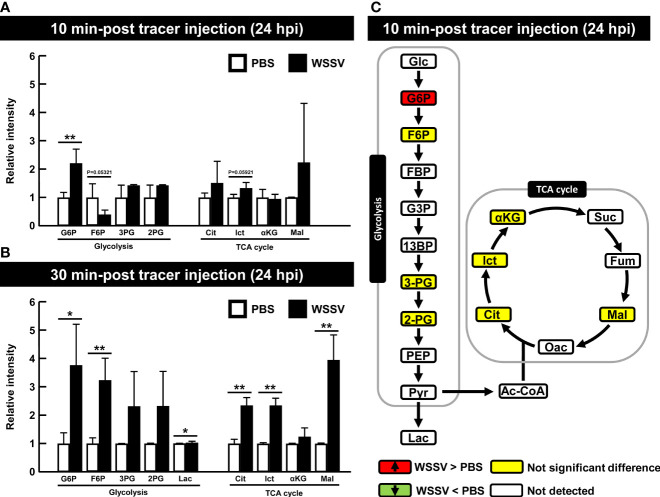
WSSV-infected hemocytes had inactivated glycolysis at the viral late stage (24 hpi) At 24 h after challenge with WSSV or PBS, shrimp were injected with [U-^13^C] glucose and hemocytes were collected after **(A)** 10 min or **(B)** 30 min of tracer injection. Metabolomic data were generated with LC-ESI-Q-TOF-MS. Fold change of each ^13^C metabolites in WSSV group compared to the corresponding ^13^C metabolites in PBS group was calculated. Each bar represents the mean ± SD. Asterisks indicate differences between WSSV and PBS groups (*p < 0.05; **p < 0.01). **(C)** Overview of changes of ^13^C metabolites in WSSV-infected shrimp (24 hpi) at 10 min post [U-^13^C] glucose injection. Changes in the WSSV group relative to the corresponding PBS control were rated as a significant increase (Red), no significant difference (Yellow), a significant decrease (Green), or not detected (White). Abbreviations are as in **Figure 1**.

At 24 hpi, although G6P was the only metabolite to be significantly increased at 10 min after tracer injection (**Figure 6A**), several other glycolytic and TCA cycle metabolites were also increased in the WSSV group at 30 min post tracer injection (**Figure 6B**). **Figure 6C** illustrates the results of **Figure 6A** and raw metabolomic data are provided in **Table S2**.

### Disruption of Glycolysis Hinders WSSV Replication

**Figure 7:**
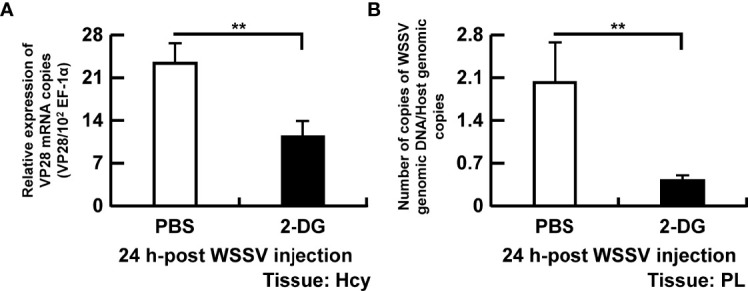
Disruption of glycolysis hinders WSSV replication. To determine the importance of glycolysis in WSSV replication, shrimp were injected with 0.5 mg/g of 2-DG twice before the WSSV challenge and the analysis done 24 h after WSSV challenge. **(A)** Gene expression of WSSV structural gene VP28 in shrimp hemocytes. **(B)** WSSV genome copy numbers quantified in pleopods. WSSV genome copy numbers was 5-fold decreased in the 2-DG injected group compared to the PBS injected group. Each bar represents the mean ± SD. Asterisks indicate differences between WSSV and PBS groups (**p < 0.01). Hcy, Hemocytes; and PL, Pleopods.

To further verify the association between glycolysis and virus replication, shrimp were treated with a glucose analogue (2-DG) and sampled at 24 hpi. The WSSV structural gene VP28 (**Figure 7A**) and WSSV genome copy numbers (**Figure 7B**) were significantly reduced in WSSV-infected shrimp pretreated with 2-DG.

## Discussion

As an obligate intracellular parasite, virus replication is highly reliant on host cell metabolism, especially glycolysis, serving as a carbon source (2, 25). In this study, 2 deoxy-D-glucose (2-DG) was used as a competitive glucose analogue to study the role of glycolysis in WSSV replication. This compound disrupted glycolysis in shrimp and impaired WSSV replication (**Figure 7**), similar to its effects on hepatitis B virus (HBV), herpesvirus, rhinovirus (RV) (26–29). As a consequence, we concluded that the glycolysis is important to WSSV replication.

Many viruses regulate glycolytic enzymes to upregulate glycolysis to facilitate viral replication (1, 2). For instance, the Avian reovirus σA protein triggers expression of glycolytic enzymes *via* HIF-1α, thereby promoting glycolysis (30). In EBV infection, protein expression of glycolytic enzymes is increased *via* EBV-encoded LMP1 to support its glycolytic addition (31). Furthermore, upregulation of glycolytic genes is reported in vertebrate virus infections, e.g., Epstein-Barr virus (LDH), herpes simplex virus (PFK), human herpesvirus 6A (HK & LDH), and influenza virus A (HK & PK) (27, 32–34). To understand regulation of glycolysis in WSSV infection, we first investigated expression of four enzymes (HK, PFK, LDH and PK) that govern the rate of glycolysis (**Figure 1**). We demonstrated that WSSV infection increased gene expression of HK and PFK (**Figures 2A**, **3A**), but not LDH or PK (**Figures 3C**, **4A**), at the viral genome replication stage (12 hpi). Liu et al. (2017) also reported that WSSV infection increased expression of HK and PFK in *Exopalaemon carinicauda* (20). Furthermore, WSSV activated glycolysis to support its replication (13, 14) and it also increased protein expression of several glycolytic enzymes, e.g., HK and PK (13). In the present study, activities of PFK, LDH and PK were elevated at the viral genome replication stage (12 hpi), whereas HK was not (**Figures 2B**, **3B, D** and **4B**). Silencing these glycolytic enzymes impaired virus replication in terms of viral structural gene (VP28) expression and viral genome copy numbers (**Figures 2D, E**, **3G, H**, **4D, E**). Additionally, compared to the PBS treated group, Luc dsRNA treatment also caused a significant decrease in WSSV mRNA expression and WSSV genome copy numbers (**Figures 2D, E**, **3G, H**, **4D, E**), perhaps due to non-specific antiviral responses attributed to dsRNAs (35). Collectively, these results demonstrated that WSSV-induced glycolysis was supported by activated glycolytic enzymes and that these glycolytic enzymes were determining factors for successful virus replication. These results were consistent with other virus-induced metabolic reprogramming, in which glycolysis is activated to support virus replication (1, 32, 36, 37). These glycolytic enzymes can produce metabolites needed for other biosynthetic pathways, e.g., nucleotides and lipids, thereby creating a favorable environment for virus replication (1–3). Interestingly, at the viral late stage, although gene expression of four glycolytic enzymes was increased in WSSV-infected shrimp, their activities were decreased (**Figures 2A, B**, **3A–D**, **4A, B**). Perhaps WSSV hinders the translation of glycolytic enzyme mRNA at this stage to facilitate the switch from viral component synthesis, which consumed biomolecule and energy, to virion morphogenesis. Ilkow et al. (2008) reported that rubella virus (RV) capsid protein inhibits protein translation by binding to poly(A)-binding protein (PABP), a host cell protein that enhances translational activities by circularizing mRNAs. This capsid-associated inhibition of translation could allow the switch from viral translation to RNA packaging into nucleocapsid (38).

Infection with WSSV did not increase HK activity at the viral genome replication stage, even though its gene expression was increased (**Figures 2A, B**). This result was not consistent with other viruses like dengue virus and EBV (6, 9), perhaps the increased glucose uptake did not overwhelm HK’s workload. Nevertheless, outcomes of dsRNA silencing emphasized the critical role of HK in glycolysis, as it catalyzes the first step of this metabolic process. Despite unchanged gene expression, LDH and PK activities were increased in WSSV-infected hemocytes at the viral genome replication stage (**Figures 3C, D**, **4A, B**). Elevated LDH and PK enzyme activity may be due to interactions between viral proteins and glycolytic enzymes, instead of classical regulation of gene expression. In that regard, interactions between host enzymes and viral proteins are used by virus to control host metabolic pathways. For example, Hepatitis C virus (HCV) and DENV use such interactions to control the metabolic rate of glycolysis, an important carbon source for virus replication (10, 11). In other studies, we are working on Yeast-2 hybrid to determine specific viral proteins that interact with these glycolytic enzymes.

Stable isotope tracing has been used to reveal distinct patterns of virus-induced metabolism (39, 40). At the WSSV genome replication stage, isotope tracing of [U-^13^C] glucose in WSSV-infected hemocytes demonstrated that glycolysis was activated. Furthermore, the glycolytic flux may have subsequently entered the TCA cycle, as the citrate (Cit) and isocitrate (Ict) were both increased 30 min after tracer injection (**Figures 5A, B**). Respiratory syncytial virus (RSV) infection caused a greater glycolytic flux being dedicated to the pentose phosphate pathway and TCA cycle (41). The fate of glycolysis differed in hepatitis B virus (HBV) infection; in that case, glucose was incorporated into the pentose phosphate pathway and hexosamine biosynthesis rather than TCA cycle (42). Based on our tracking, lactate did not increase at the same time point during WSSV infection (**Figure 5A**), which contrasts with the previous result. Using a stable glutamine isotope, He et al. (2019) revealed that glutamine contributed to production of lactate *via* oxidative glutamine metabolism, suggesting that lactate produced during WSSV infection might be derived from glutamine (15). However, it was not possible to conclude that the WSSV-induced glycolysis solely contributed to the TCA cycle, as the discrepancy between results could be explained by either removal of hemolymph (equivalent to human blood plasma) during the experiment or the usage of lactate as a fuel source. Lactate, a metabolic waste product, must be discarded from eukaryotic cells to prevent detrimental cytosol acidification and promote glycolysis, given that a build-up of cytosolic lactate downregulates the rate-limiting enzymes, HK and PFK (43, 44). In a previous study, carbon from lactate was incorporated into lipids *via* the TCA cycle in human lung cancer and cervical cancer cells (45). Furthermore, lactate dehydrogenase B (LDHB) is localized in the inner mitochondrial membrane, which may facilitate the conversion of lactate to pyruvate (45). In WSSV infection, the lack of lactate at 12 hpi might have been due to a high demand for it for the TCA cycle, where citrate can be readily produced and then used to generate Acetyl-CoA for WSSV-induced lipogenesis. Whether some shrimp LDH is localized in mitochondria is unknown; however, based on the example from cancer cells, lactate may be metabolized in WSSV-infected hemocytes, although further work is needed to clarify it.

Citrate, a TCA cycle intermediate, was greatly increased at 12 hpi and at 30 min after tracer injection (**Figure 5B**). It is a metabolite that can be transported out of mitochondria, where it is cleaved by ATP citrate lyase to generate cytosolic acetyl-CoA, a crucial metabolite for virus replication because it can serve as a carbon backbone for lipid synthesis or acetylation of viral protein (46, 47). Our previous study on WSSV-induced glutaminolysis also demonstrated contributions of glutamine to the TCA cycle, causing accumulation of citrate at the 12 hpi (15). As two major carbon input pathways, glycolysis and glutaminolysis were implicated in replication of various virus, including Marek’s disease virus, Herpevirus and HCV (4, 7, 48). Taken together, both glycolysis and glutaminolysis were increased and perhaps entered the TCA cycle at the WSSV genome replication stage.

At the late stage of virus replication, despite no significant changes in metabolites in the first 10 min after tracer injection, various metabolites were increased at 30 min after tracer injection. The glycolytic pathway required more time to metabolize the tracer, resulting in a slow glycolytic rate (**Figure 6**). We inferred that glycolysis was more active at the viral genome replication stage than at the viral late stage, even though lipogenesis, which may provide fatty acids for the WSSV viral envelope, is triggered at the viral late stage (16). Perhaps metabolites required for lipogenesis are generated earlier. Human cytomegalovirus (HCMV) rerouted the glycolytic flux into fatty acid synthesis (46). Perhaps WSSV uses the same strategy as HCMV to provide metabolites for lipogenesis. Future studies are needed to determine whether Acetyl-CoA, an initial substrate for lipogenesis, is generated from citrate outside mitochondria at the early time point, but subsequently used for lipogenesis at viral late stage.

In conclusion, this study demonstrated WSSV activated glycolysis *via* upregulating shrimp glycolytic enzymes. The increased glycolysis then may contribute to nucleotide synthesis *via* the pentose phosphate pathway (13), and possibly TCA cycle (**Figure 5**) and lipogenesis by increasing availability of Acetyl-CoA. Intermediates within glycolysis may also serve as substrate for other biosynthetic pathways for virus replication. However, several questions remained to be answered. For example, the factors or components that mediates communication between WSSV and the glycolysis pathway remained to be elucidated.

## Data Availability Statement

The original contributions presented in the study are included in the article/**Supplementary Material**. Further inquiries can be directed to the corresponding author.

## Author Contributions

YN, C-YT, and S-TH designed and performed *in vivo* animal experiments and analyzed data. D-YL performed LC-ESI-MS-based isotopic labeled metabolomic analysis. C-HL provided shrimp for animal experiment. YN wrote the manuscript. H-CW conceived the idea, designed the research, discussed data, and supervised this work. All authors contributed to the article and approved the submitted version.

## Funding

This study was supported financially by the Ministry of Science and Technology, Taiwan (MOST 108-2314-B-006-096-MY3; MOST 110-2634-F-006-019).

## Conflict of Interest

The authors declare that the research was conducted in the absence of any commercial or financial relationships that could be construed as a potential conflict of interest.

## Publisher’s Note

All claims expressed in this article are solely those of the authors and do not necessarily represent those of their affiliated organizations, or those of the publisher, the editors and the reviewers. Any product that may be evaluated in this article, or claim that may be made by its manufacturer, is not guaranteed or endorsed by the publisher.
