# Supplementary material for: White Spot Syndrome Virus Triggers a Glycolytic Pathway in Shrimp Immune Cells (Hemocytes) to Benefit Its Replication
Source: Front Immunol. 2022 Jul 4;13:901111. doi: 10.3389/fimmu.2022.901111 (PMC9289281; doi:10.3389/fimmu.2022.901111)
Supplement: Supplementary file 1 [file DataSheet_1.docx]

Supplementary Material

# Supplementary Table

## Table S1. Quantitative changes in the glycolytic metabolites in WSSV-infected shrimp hemocytes at 12 hpi as determined by [U-^13^C] glucose tracing.

| **Metabolites** | | **10 min** | | | **30 min** | | |
| --- | --- | --- | --- | --- | --- | --- | --- |
|  |  | **PBS** | **WSSV^a^** | **Fold change^b^** | **PBS** | **WSSV^a^** | **Fold change^b^** |
| **Glycolysis** | **Glucose 6-phosphate** | 458.476  ± 181.761 | 2958.722  ± 145.064 | 6.453 | 93.206  ± 23.321 | 2146.026  ± 102.198 | 23.025 |
|  | **Fructose 6-phosphate** | 95.945  ± 7.900 | 761.083  ± 151.832 | 7.933 | 52.170  ± 40.486 | 535.903  ± 106.753 | 10.272 |
|  | **2-Phosphoglycerate^c^** | 618.790  ± 22.584 | 1306.375  ± 99.077 | 2.111 | 604.990  ± 5.935 | 1198.258  ± 438.025 | 1.981 |
|  | **3-Phosphoglycerate^c^** | 618.790  ± 22.584 | 1306.375  ± 99.077 | 2.111 | 604.990  ± 5.935 | 1198.258  ± 438.025 | 1.981 |
|  | **Lactate** | 137.931  ± 0.000^d^ | 115.040 ±4.540 | 0.834 | - | - | - |
| **TCA cycle** | **Citrate** | 2128.997  ± 252.954 | 2394.981  ± 105.994 | 1.125 | 936.524  ± 44.582 | 4408.194  ± 181.923 | 4.707 |
|  | **Isocitrate** | 604.503  ± 231.609 | 1564.944  ± 156.538 | 2.589 | 568.421  ± 42.934 | 2185.750  ± 333.558 | 3.845 |
|  | **α-Ketoglutarate** | 137.931  ± 0.000^d^ | 114.757  ± 6.316 | 0.832 | 137.931  ± 0.000^d^ | 120.452  ± 16.179 | 0.873 |
|  | **Succinate** | - | - | - | 311.186  ± 300.087 | 111.111  ± 0.000^d^ | 0.357 |
|  | **Malate** | 144.874  ± 12.025 | 353.320  ± 299.129 | 2.439 | 137.931  ± 0.000^d^ | 774.394  ± 937.036 | 5.614 |

^a^Red and green indicate significant up- and down-regulation, respectively. Yellow indicates no significant change.

^b^Fold change: WSSV / PBS

^c^Metabolomic data derived from 2-Phosphoglycerate and 3-Phosphoglycerate cannot be separated since the chemical structure of them is almost identical. In glycolysis, phosphoglycerate mutase catalyzes the conversion from 3-phosphoglycerate to 2-phosphoglycerate, in which the phosphate group is relocated from an end carbon to a central carbon.

^d^The value of the signal generated from these metabolomic samples was lower than the limit of quantification, therefore, lowest value that can be quantified is assigned to these samples and used for the calculation.

## Table S2. Quantitative changes in the glycolytic metabolites in WSSV-infected shrimp hemocytes at 24 hpi as determined by [U-^13^C] glucose tracing.

| **Metabolites** | | **10 min** | | | **30 min** | | |
| --- | --- | --- | --- | --- | --- | --- | --- |
|  |  | **PBS** | **WSSV^a^** | **Fold change^b^** | **PBS** | **WSSV^a^** | **Fold change^b^** |
| **Glycolysis** | **Glucose 6-phosphate** | 298.575  ± 51.721 | 659.694  ± 151.520 | 2.209 | 231.687  ± 87.858 | 873.545  ± 334.743 | 3.770 |
|  | **Fructose 6-phosphate** | 188.850  ± 88.435 | 77.802  ± 27.519 | 0.412 | 96.671  ± 20.839 | 314.123  ± 73.299 | 3.249 |
|  | **2-Phosphoglycerate^c^** | 267.977  ± 114.941 | 384.472  ± 3.259 | 1.435 | 125.000  ± 0.000^d^ | 289.406  ± 152.691 | 2.315 |
|  | **3-Phosphoglycerate^c^** | 267.977  ± 114.941 | 384.472  ± 3.259 | 1.435 | 125.000  ± 0.000^d^ | 289.406  ± 152.691 | 2.315 |
|  | **Lactate** | - | - | - | 125.000  ± 0.000^d^ | 130.143  ± 4.301 | 1.041 |
| **TCA cycle** | **Citrate** | 1273.259  ± 205.136 | 1921.776  ± 978.907 | 1.509 | 1750.312  ± 278.954 | 4116.574  ± 466.837 | 2.352 |
|  | **Isocitrate** | 586.290  ± 66.463 | 771.415  ± 131.748 | 1.316 | 674.229  ± 31.239 | 1591.915  ± 167.105 | 2.361 |
|  | **α-Ketoglutarate** | 154.440  ± 43.740 | 143.870  ± 27.791 | 0.932 | 125.000  ± 0.000^d^ | 157.040  ± 38.004 | 1.256 |
|  | **Malate** | 125.000  ± 0.000^d^ | 280.026  ± 263.906 | 2.240 | 125.000  ± 0.000^d^ | 496.902  ± 107.384 | 3.975 |

^a^Red and green indicate significant up- and down-regulation, respectively. Yellow indicates no significant change.

^b^Fold change: WSSV / PBS

^c^Metabolomic data derived from 2-Phosphoglycerate and 3-Phosphoglycerate cannot be separated since the chemical structure of them is almost identical. In glycolysis, phosphoglycerate mutase catalyzes the conversion from 3-phosphoglycerate to 2-phosphoglycerate, in which the phosphate group is relocated from an end carbon to a central carbon.

^d^The value of the signal generated from these metabolomic samples was lower than the limit of quantification, therefore, lowest value that can be quantified is assigned to these samples and used for the calculation.
